# 40th Anniversary Virtual Issues: Genomic Innovations in Extremophiles

**DOI:** 10.1093/molbev/msae159

**Published:** 2024-08-16

**Authors:** Casey L McGrath

To celebrate the 40th anniversary of the first issue of *Molecular Biology and Evolution*, the Society for Molecular Biology and Evolution (SMBE) journals are publishing a series of virtual issues in 2024. These virtual issues contain selected papers published in the society's journals, *Molecular Biology and Evolution* and *Genome Biology and Evolution*. Each virtual issue is accompanied by a Perspective that highlights the historic and contemporary contributions of our journals to a specific topic in molecular evolution.

August's Perspective, titled “Lessons from extremophiles: genomic innovations and functional adaptations across the eukaryotic tree of life”, appears in *Genome Biology and Evolution* and was written by H.B. Rappaport and Angela M. Oliverio. The accompanying virtual issues—one for *Molecular Biology and Evolution* and one for *Genome Biology and Evolution*—include noteworthy papers on Genomic Innovations in Extremophiles published in the two journals and can be found at SMBE’s 40th anniversary site.

